# Intestinal responses in pacu (*Piaractus mesopotamicus*) exposed to fasting and refeeding nutritional management

**DOI:** 10.1002/ar.25683

**Published:** 2025-05-15

**Authors:** Karine Nathiele Nogueira Farias, André Luiz do Nascimento Silva, Marco Shizuo Owatari, Sabrina Fuzer Gonçalves, Robson Andrade Rodrigues, Cristiane Meldau de Campos, Lilian Franco‐Belussi, Carlos Eurico Fernandes

**Affiliations:** ^1^ Programa de Ciência Animal da Faculdade de Medicina Veterinária e Zootecnia Universidade Federal de Mato Grosso do Sul Campo Grande Mato Grosso do Sul Brazil; ^2^ Laboratório de Patologia Experimental—LAPEx Instituto de Biociência, Universidade Federal de Mato Grosso do Sul Campo Grande Mato Grosso do Sul Brazil; ^3^ Departamento de Aquicultura Universidade Federal de Santa Catarina—UFSC Florianópolis Santa Catarina Brazil; ^4^ Unidade Universitária de Aquidauana Universidade Estadual de Mato Grosso do Sul—UEMS Camisão Mato Grosso do Sul Brazil

**Keywords:** aquaculture, fish farming, food deprivation, intestine, mucus cells, stereology

## Abstract

Fasting is a practice in the aquaculture industry that aims to reduce feed costs. This practice can affect the gastrointestinal system of fish. The intestine plays a pivotal role in the nutrition and overall health of fishes. The present study sought to evaluate the effects of fasting and refeeding on body weight, intestine somatic index (ISI), intestinal histometry (area, height, and thickness of the villi), and goblet cell volume (acidic and neutral mucins) of pacu (*Piaractus mesopotamicus*). To this end, the effects of 10, 20, and 30 days of fasting and 15 and 50 days of refeeding were evaluated. The results demonstrated that fasting resulted in notable alterations in all assessed parameters. The body weight and ISI of fasted pacu were significantly reduced compared to those of the control group. The levels of acidic and neutral mucins were elevated after 10 days of fasting, whereas the area and height of the villi decreased after 20 days of fasting. Following the 15‐day refeeding period, pacu exhibited an increase in body weight and ISI. However, a 50‐day refeeding period was required to observe an increase in villus height, which differed from that in the control group. After 50 days of refeeding, the fish exhibited intestinal conditions that were restored to the levels observed in the control specimens. Feed deprivation alters intestinal biometry and histomorphology. However, the effects of fasting were attenuated and even improved with subsequent refeeding. These results suggest that a fasting/refeeding management strategy is appropriate for pacu farming.

## 1 INTRODUCTION

Food deprivation is a phenomenon that many fish encounter during their life cycle due to the decrease in food available in their environment. This practice of reducing food intake or fasting is used in the aquaculture industry to reduce feed costs without harming animals (Favero et al., 2020; Rueda et al., 1998; Urbinati et al., 2014). A sustainable production system with biofloc technology reduced by up to 5% dietary protein of *Piaractus mesopotamicus* did not affect growth, as the biofloc serves as a complementary food source (Sgnaulin et al., 2021). *P. mesopotamicus* is native to Brazil and its cultivation has increased in the aquaculture industry.

The effects of fasting in *P. mesopotamicus* are described as changes in the structural composition of the liver, as well as a decrease in the size of hepatocytes, glucose, and triglycerides, and an increase in cholesterol (Farias et al. 2024). However, the effects of fasting and refeeding on the intestines of *P. mesopotamicus* have not been described. The anterior intestinal section of *P. mesopotamicus* is associated with nutritional absorption and digestion and exhibits an elevated villus height, as well as an increased number of mucous and granulocytic cells compared to other intestinal sections (Bellinate et al., 2023). However, owing to variations among species, the characteristics of the gastrointestinal tract may diversify functionally and morphologically in aquacultured fish (Bakke et al., 2010; Zhao et al., 2019). Fasting can cause structural changes and metabolic adaptations in fish intestines (Gaucher et al., 2012; Sun et al., 2020) such as decreased intestinal villus length and mucosa/submucosa thickness, as well as a reduction in the number of goblet cells (Shen et al., 2021). These changes probably occur because of the absence of feeding stimulation, leading to immunological and/or inflammatory responses in the intestine, which can be examined by organ histometry (Day et al., 2014; Fabregat et al., 2011; Zhao et al., 2021).

During fasting, fish exhibit increased appetite and reduced body weight. In contrast, fish can increase their feed intake during refeeding and present increased growth compared with continuously fed animals (Fang et al., 2021; Favero et al., 2020; Silva et al., 2019). The ability of a fish to undergo periods of fasting followed by refeeding is an example of digestive flexibility. This allows fish to optimize their nutrients while reducing the maintenance costs associated with their digestive systems. During fasting, the intestines exhibit significant plasticity, which can result in substantial atrophy. However, even after prolonged fasting, the intestines demonstrate remarkable resilience and may recuperate rapidly during refeeding (Zaldúa & Naya, 2014).

Morphofunctional changes throughout the digestive tract characterize the process of nutrient digestion in fish. These changes are driven by various structures, including villi, microvilli, defense cells, and digestive enzymes, which interact with a robust microbiological network to contribute to the overall absorption mechanisms within the intestinal tract (Bakke et al., 2010; Genten et al., 2009; Veiga et al., 2020). The mucous epithelial layer is populated by two distinct cell types: columnar cells, which are essential for the digestion and absorption of nutrients, and goblet cells, which secrete mucus and glycoproteins and assist in enzymatic digestion and antimicrobial activity (Bellinate et al., 2023; Sherif et al., 2020). Therefore, the digestive tract of fish serves as a valuable indicator of their health status in aquaculture (Bellinate et al., 2023). However, the cultivation of species such as *P. mesopotamicus* is currently lacking in terms of research on the morphophysiological responses of the intestine to fasting, which is defined as the period of total feed deprivation.

The mucosal layer covers the intestinal contact surfaces. The composition, structure, and thickness of the mucus and the characteristics of the mucosal layer may vary depending on the specific mucosal region and the prevailing physiological, immunological, or environmental conditions. Goblet or mucous cells are distributed along the mucous layer of the intestine and produce different types of mucus (Castro & Tafalla, 2015). The mucus is composed of water and glycoproteins (mucins), which form a gel structure containing various humoral immune factors with activities against pathogen infiltration. Mucins are the major structural components of the mucus layer, which contains various humoral immune factors and provides viscoelastic and protective properties (Salinas et al., 2022).

The present study aimed to evaluate the plasticity of the anterior portion of the intestinal tract of *P. mesopotamicus* following distinct periods of fasting and refeeding by measuring morphological structural responses and goblet cell volume.

## 2 MATERIALS AND METHODS

The experimental procedures were approved by the Ethics Committee on the Use of Animals (CEUA) of the Federal University of Mato Grosso do Sul (UFMS; protocol no. 834/2017), and were in accordance with established ethical principles.

### 2.1 Experimental design

Juvenile *P. mesopotamicus* (*n* = 120; 47.7 ± 9.2 g and 10.6 ± 0.8 cm standard length) were acclimatized to the experimental conditions for 15 days before treatments (i.e., fasting and refeeding) under controlled temperature (approximately 26–28°C) and natural lighting (12 h dark, 12 h light), constant aeration, and physical and biological filtration. Fish were fed (*ad libitum*) twice daily with an extruded commercial feed for omnivorous fish (Guabi‐Pirá® [approximately 4–6 mm], 8% moisture, 32% crude protein, 6.5% ether extract, 7% crude fiber, and 10% mineral matter). Water quality parameters (temperature, dissolved oxygen, pH, and electrical conductivity) were measured twice daily using a SANXIN SX751 portable meter. After acclimation, initial biometrics (weight and standard length) were measured to determine the biomass and amount of feed (5% of live weight) to be administered. The fish were randomly distributed into six 140 L experimental units (20 fish per unit) and divided into two treatments with three replicates each.

The fasting treatment was performed for 30 days with a total of 72 fish. During fasting, 12 fish were analyzed at each experimental period after 10, 20, and 30 days. For each experimental period, we evaluated 12 fish from the control group that were fed ad libitum.

The refeeding treatment began with an additional 24 fish that had been fasted for 30 days. This treatment consisted of refeeding the animals. Twelve animals each were analyzed after 15 and 50 days of refeeding treatment, respectively. For this treatment, the control group comprised 24 fish that were fed ad libitum and were in the acclimation period. This is the standard procedure for maintaining control groups used in fish management and production to determine whether the animals continued to gain the same weight after fasting. Therefore, we used this feeding protocol to evaluate the morphological characteristics of the intestine.

### 2.2 Intestinal samples, intestine somatic index, and intestinal histology

After each experimental period, all animals in the experimental group were euthanized with an overdose of eugenol anesthetic (450 mg L^−1^) as described by Rotili et al. (2012) for intestinal analyses. Immediately after euthanasia, the specimens (*n* = 12 per experimental period) were weighed (g), and the intestine was removed to determine the intestine somatic index (ISI %) using the following equation:

ISI(%)=[intestine weight (g)/body fish weight (g)]× 100.

For histological analysis, we randomly selected six fish per experimental period, and the anterior portion of the intestine was removed and dissected, as described by Bellinate et al. (2023). The samples were fixed in a 10% buffered formalin solution (pH 7.2) for 24 h. Subsequently, the samples were processed into routine paraffin histology and sectioned into 4‐μm sections. The sections were stained with hematoxylin–eosin (H&E) and histochemical reactions were performed to quantify the volume of acid mucus cell secretion (Alcian blue pH 2.5, AB) and neutral mucus cell secretion (periodic acid‐Schiff [PAS]).

### 2.3 Intestinal histometry

For intestinal histometric analyses, six fishes per experimental period were analyzed in fasting and refeeding conditions. Furthermore, slides stained with H&E were photographed with an OptCam® camera (LOPT14003, RGB 4096 × 3286 pixels) coupled to a high‐performance vertical optical microscope (OptCam‐O500R®) at 100× and 400× magnification. Seven images of each fish were obtained from six specimens per experimental group (42 images per group). In each image, villi area (μm^2^) and height (μm), and intestinal mucosa thickness (μm) were estimated in three random fields. The average values obtained for each image were considered. The Motic Image Plus 2.0 software was used for these analyses.

### 2.4 Volume of acidic and neutral mucus cells

The volume of the mucus cells was determined using slides stained with AB (pH: 2.5) and PAS as described by Carson and Hladik (2009). Forty mucus cells were measured in five randomly selected villi from three randomly selected fish at each experimental period. The average of the largest and smallest cell diameters was used to calculate cell volume using the following formula: *V* = (4/3*πr* ^3^), where *V* refers to the volume (μm^3^) and *r* refers to the radius value. The volume was calculated by assuming that the cells were approximately spherical. Motic Image Plus 2.0 software was used to perform the measurements.

### 2.5 Statistical analysis

Prior to statistical analysis, data regarding normality (Kolmogorov–Smirnov test) and homogeneity of variances (Levene test) were analyzed. All data were normally and homogeneously distributed. Time‐fasting and refeeding effects between the control and exposure groups were compared using a generalized linear mixed model of analysis of variance (ANOVA), followed by pairwise comparisons using the least significant difference test. SPSS 23.0 (IBM®) software was used for these analyses. Box plots were created for the mean, 25th and 75th quartiles, minimum and maximum values, and interquartile intervals. All analyses were performed at a significance level of 5%.

## 3 RESULTS

**FIGURE 1:**
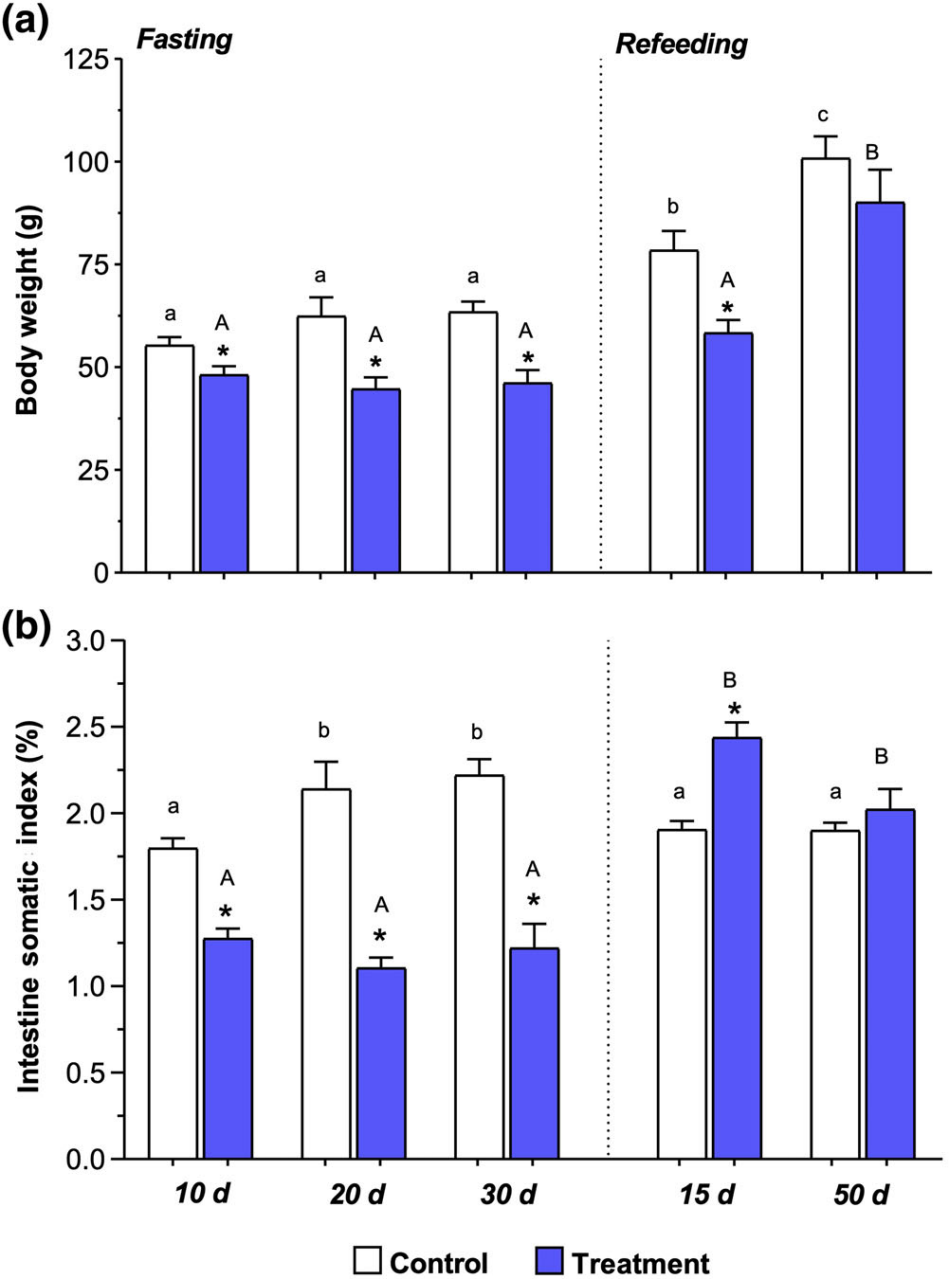
Mean (± Standard Error of the Mean (SEM)) of biometric data of pacu (*Piaractus mesopotamicus*) after 10, 20, or 30 days of fasting and 15 or 50 days of refeeding. In (a), body weight. In (b), intestine somatic index (%). (*) indicates a significant difference (*p* < 0.05) between groups in the same experimental period (control × treatment). Lowercase letters indicate a significant difference between control groups. Capital letters indicate a difference between the fasting and refeeding periods. All analyses were performed with a significance level of 5% (*p* < 0.05).

Body weight and ISI of fasted *P. mesopotamicus* were significantly reduced (*p* < 0.05) at all sampling periods (10, 20, and 30 days) compared to those of the control group. After 15 days of refeeding, the body weight remained significantly lower (*p* < 0.05) in the refeeding group than in the control group. In contrast, after 50 days of refeeding, the body weight did not show a significant difference (*p* > 0. 05) between the treatment and control fish; that is, the fasted‐then‐refed fish had weights equivalent to those that were fed continuously. The ISI of the treated fish increased significantly (*p* < 0.05) after 15 days of refeeding, and was higher than that of the control group and fish that were fasted for 10, 20, and 30 days. However, after 50 days of refeeding, no significant differences (*p* > 0.05) were observed in ISI between the refeeding and control groups (Figure 1).

### 3.1 Intestinal anatomy and histology

**FIGURE 2:**
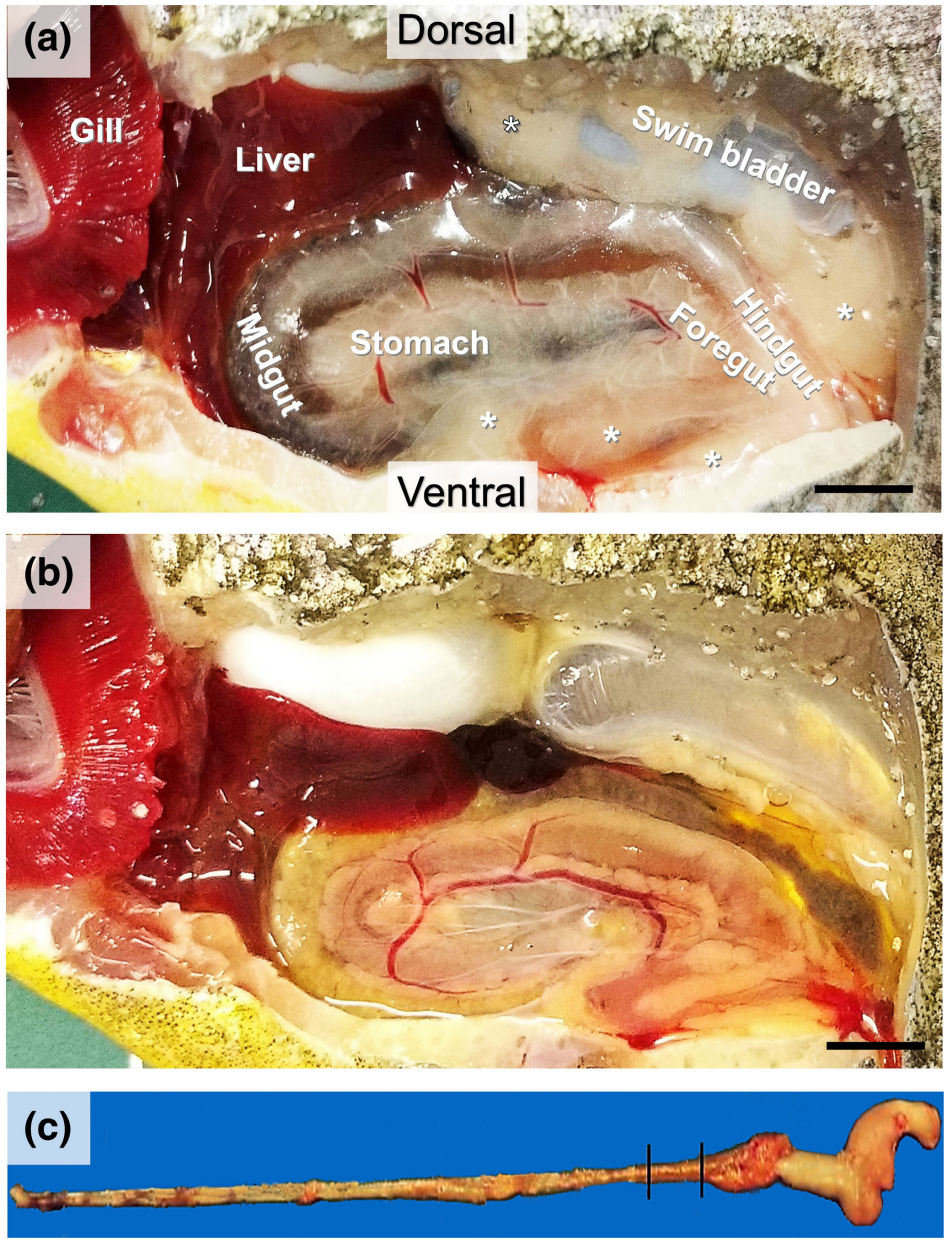
Lateral view of gross anatomy of the intestinal tract of *Piaractus mesopatomicus* in the control group (a) showing symbiotic relationship between organs, and after 30 days of fasting (b). Visceral fat deposits with predominance in the control specimen (*). Isolated digestive tube demonstrating the analyzed fragment of the anterior intestine (c).

**FIGURE 3:**
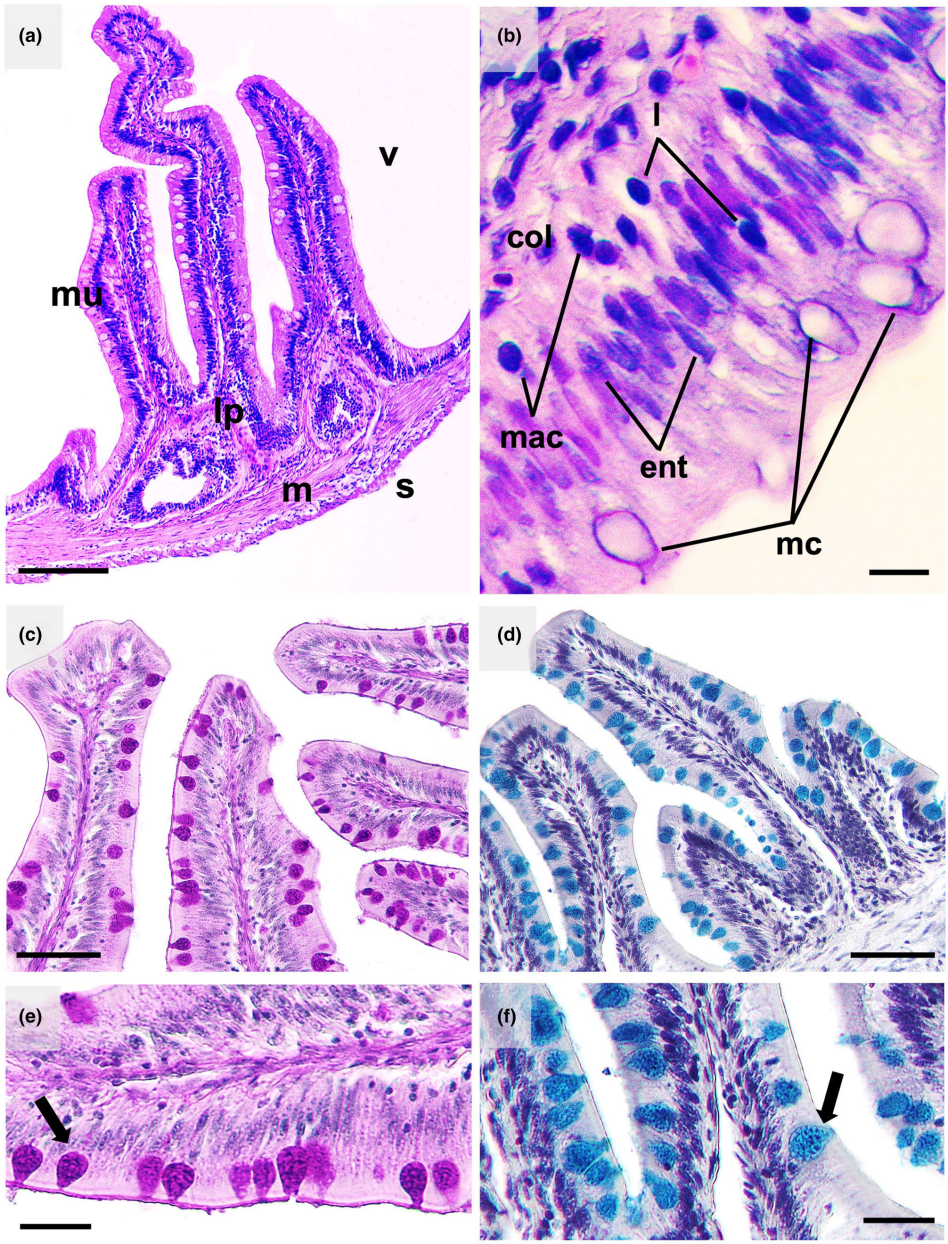
Intestinal histological sections of pacu (*Piaractus mesopotamicus*). In (a), intestinal villus, mucosa (mu), lamina propria (lp), muscular layer (m), and serosa (s), stained with hematoxilin‐eosin. In (b), intestinal mucosa, mucus cells (mc), macrophages (mac), enterocytes (ent), lymphocytes (l), and collagen (col). In (c) and (e), mucus cells are stained positively for neutral mucins by Periodic Acid‐Schiff staining. In (d) and (f), mucus cells are stained positively for acidic mucins by Alcian Blue staining; arrows indicate cytoplasmic granulation. Scale bars: (a) 100 μm; (b) 5 μm; (c) and (d) 50 μm; and (e) and (f) 20 μm.

The foregut of *P. mesopotamicus* is located dorsal to the stomach and ventral to the hindgut (Figure 2). The intestinal wall of *P. mesopotamicus* is composed of four layers, similar to that of other vertebrates: the mucosa, submucosa, muscular, and serosa (Figure 3a). The mucosa is folded into villi with microvilli, which increase the surface area available for nutrient absorption. Columnar cells in the mucous epithelium are characterized as enterocytes or absorptive cells, which are located below the goblet cells, in addition to granulocytes (lymphocytes), agranulocytes, and macrophages, which are responsible for secretion and absorption (Figure 2b). The submucosa is located below the lamina propria and is formed by loose connective tissue irrigated by blood vessels that irrigate the mucosa, glycoproteins, and collagen fibers. The serosa is characterized by loose connective tissue internally, and externally by a layer of squamous epithelial cells. In the intestinal epithelium, the mucus cells were stained for neutral (PAS) (Figure 3c,e) and acidic mucins (AB) (Figure 3d,f).

### 3.2 Intestinal histometry

**FIGURE 4:**
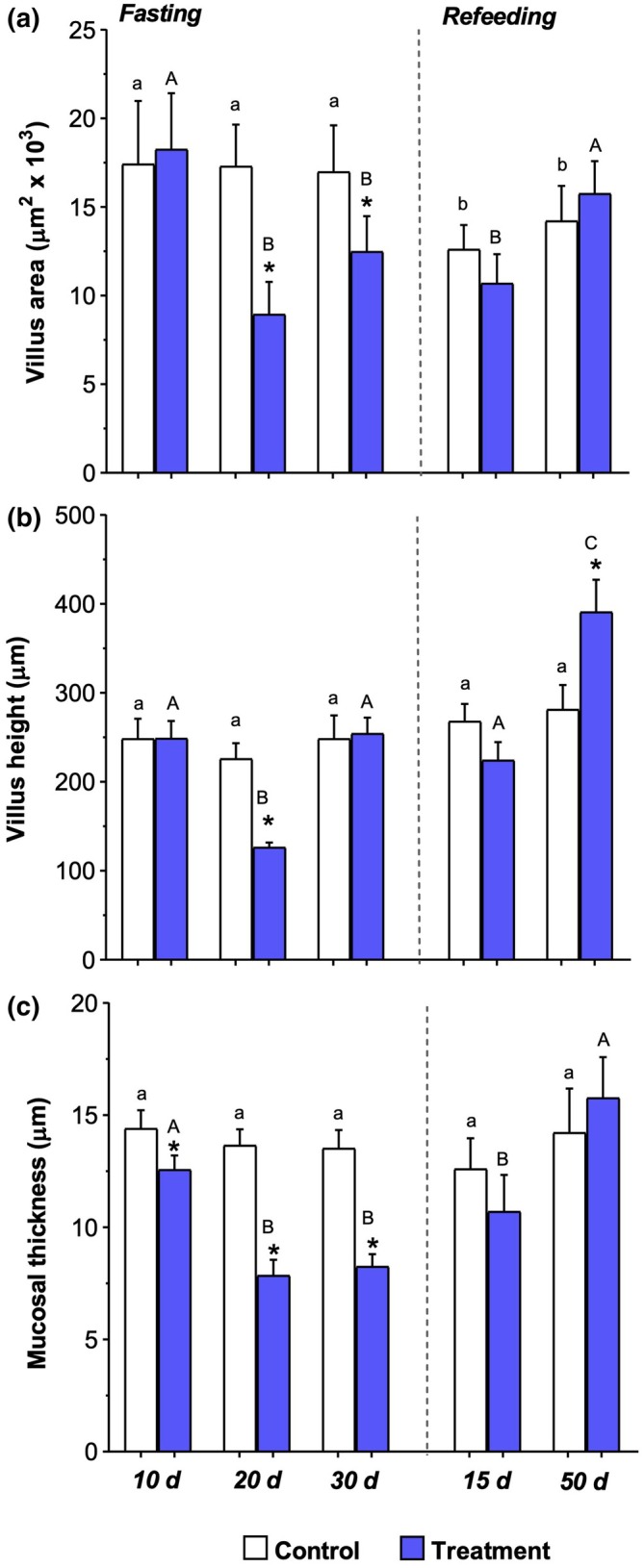
Mean (± SEM) of the intestinal histomorphometry measurements of pacu (*Piaractus mesopotamicus*) after 10, 20, or 30 days of fasting and 15 or 50 days of refeeding. In (a), villi area (μm^2^). In (b), villi height (μm). In (c), villi thickness (μm). (*) indicates a significant difference between groups in the same period (control × treatement). Lowercase letters indicate a significant difference between control groups. Capital letters indicate a significant difference between the experimental periods. All analyses were performed with a significance level of 5% (*p* < 0.05).

The area and height of the intestinal villi were significantly reduced (*p* < 0.05) in 20‐day‐fasted *P. mesopotamicus* compared to those in the control groups, as well as after 10 days of fasting (Figure 4a,b). After 50 days of refeeding, villus heights increased significantly (*p* < 0.05), being higher than that in the control and 15‐day refeeding group, and different from the other fasting periods.

After 20 and 30 days of fasting, there was a significant reduction (*p* < 0.05) in mucosal thickness compared to the control group and in fish after 10 days of fasting (Figure 4c). After 50 days of refeeding, a significant increase (*p* < 0.05) in mucosal thickness was observed in both refeeding periods compared with the 20‐ and 30‐day fasting periods.

No significant difference was observed (*p* > 0.05) in the mucosal thickness between the refeeding and control groups after 50 days of refeeding, indicating recovery of the intestinal structure.

### 3.3 Volume of acidic and neutral goblet cells

**FIGURE 5:**
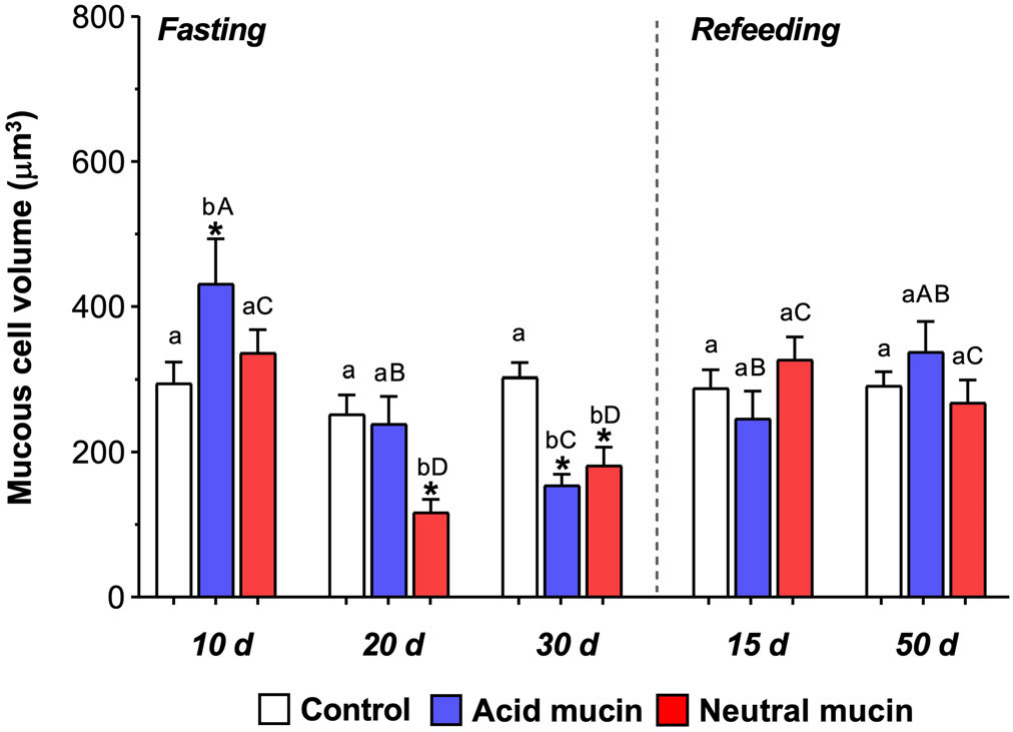
Mean (± SEM) of the volume of the acidic (stained with Alcian Blue) and neutral (stained with periodic acid‐Schiff) mucus cells of pacu (*P. mesopotamicus*) after 10, 20, or 30 days of fasting and 15 or 50 days of refeeding. (*) indicates significant differences between groups in the same experimental period (control × treatment). Capital letters indicate a significant difference between mucins within the same experimental period (acidic × neutral); lowercase letters indicate a significant difference between each mucin across different experimental periods. All analyses were performed with a significance level of 5% (*p* < 0.05).

The volume of goblet cells producing acid mucins significantly increased (*p* < 0.05) after 10 days of fasting and significantly decreased (*p* < 0.05) after 30 days of fasting compared with the control group. In contrast, the volume of goblet cells producing neutral mucins was significantly lower (*p* < 0.05) after 20 and 30 days of fasting than in the control group. After refeeding, the goblet cell volume was restored, not differing significantly (*p* > 0.05) between the refeeding and control groups (Figure 5).

## 4 DISCUSSION

Fasting negatively affected body weight, ISI, villus area and height, mucosal thickness, and mucus cell volume; however, all parameters recovered with refeeding for up to 50 days. Feeding strategies consisting of food deprivation or restriction followed by refeeding have gained prominence over the years in aquaculture, enabling a reduction in production costs associated with feeding without impairing animal growth performance owing to compensatory growth until the harvest period (Favero et al., 2018; Hvas et al., 2022; Py et al., 2022; Reigh et al., 2006). However, before extending this practice to aquaculture, the physiological costs associated with fasting periods and compensatory growth after refeeding (Py et al., 2022) must be examined. In the present study, pacu (*P. mesopotamicus*) exhibited reduced weight and ISI during feed deprivation. At this challenging stage, metabolic activities were probably reduced, which minimized the energy costs for physiological maintenance. Energy reserves such as lipids present in the intestine were likely mobilized, leading to the observed reduction in intestinal weight. Morphologically, the changes observed in the intestinal tract of *P. mesopotamicus* demonstrated that the fasting period abruptly ceased the intestinal stimulation related to digestion and feed absorption. In contrast, growth recovery by the end of the 50 days of refeeding and accelerated body weight gain, reflected in the final growth and increased ISI, indicating optimal compensatory growth.

The absence of a feeding stimulus influenced the reduction in villus area, height, and thickness during fasting. After 20 and 30 days of fasting, both villus area and height decreased, and after 10 days of fasting, mucosal thickness decreased in *P. mesopotamicus*. Similar effects of food deprivation were observed in *Rhamdia quelen* with decreased epithelial area of the intestine (Hernández et al., 2018). These variations are expected to be due to intestinal plasticity, considering that intestinal morphology can vary between specimens (Zaldúa & Naya, 2014). However, the physiological mechanisms underlying this process are not entirely understood (Py et al., 2022). Similarly, the reduction in mucosal thickness was probably influenced by the inactivation of absorptive cells and atrophy of enterocytes due to the absence of feeding stimulation. Refeeding increased the height and thickness of the mucosa, providing better conditions for the growth of villi and the possibility of enterocyte hypertrophy (Laczynska et al., 2020), increasing absorption and better use of nutrients. Enterocytes play a fundamental role in this process by ensuring the absorption of ions, water, nutrients, vitamins, and salts (Dawood, 2021), thereby providing good nutritional indicators for understanding the intestinal development of fish (Ostaszewska et al., 2006).

Similar to the present study, other studies have described intestinal histometric changes in fish after food deprivation. Zeng et al. (2012) noted that the morphology and structure of the gastrointestinal tract of catfish (*Silurus meridionalis*) were significantly negatively regulated, as indicated by a decrease in the thickness of the stomach and intestinal mucosa, density of goblet cells, and surface area of microvilli, implying that fasting greatly impaired the digestive and absorptive functions of the gastrointestinal tract. Additionally, when deprived of feed, *S. meridionalis* can tolerate severe periods of starvation and downregulate digestive tract functions using adaptive physiological and biochemical strategies.

In contrast, Shen et al. (2021) reported that the intestinal function of mudskippers (*Boleophthalmus pectinirostris*) recovered faster after fasting under semiaquatic conditions than under underwater conditions. However, the length of the intestinal villi and thickness of the intestinal mucosa/submucosa decreased significantly during fasting; however, they were thinner in the semiaquatic environment. Whereas the constitutive mRNA expression of glucose, long‐chain fatty acids, and amino acid transporters increased dramatically in the intestines of *B. pectinirostris* under semiaquatic conditions after fasting. Furthermore, fasting induces pro‐inflammatory responses in the early and intermediate stages and anti‐inflammatory responses in the intermediate and late stages, indicating its pivotal role in modulating intestinal structure and function.

Different dietary levels of proteins and lipids can increase the height and width of the villi and thickness of the muscle layer (Wu et al., 2020). Feed deprivation reduces the metabolic stimulus and consequently decreases the intestinal absorption function, reducing the size of enterocytes and the mucosal layer. The increased volume of acidic mucins (stained with AB) compared with that of neutral mucins (stained with PAS) is related to inflammatory stimuli in the intestinal microbiota triggered by the immune system. Previous studies have suggested that fasting in fish alters the intestinal microbiota due to stress. It likely stimulates both digestive and immunological enzymatic activities, suggesting an increase in the mRNA expression of glucose, lipids, and proteins and triggering an increase in the expression of pro‐inflammatory cytokines at the beginning of fasting (Shen et al., 2021).

Given this scenario, acidic goblet cells perform crucial functions, as they are responsible for protecting the epithelial mucosa against bacterial activity and possible biochemical and mechanical damage (Bosi & Dezfuli, 2015; Matos et al., 2017), while neutral goblet cells are responsible for secreting sulfated substances that regulate the acid content of the intestinal lumen (Domeneghini et al., 2005), supporting the digestion and emulsification of the feed bolus, in addition to protecting the mucous epithelium with a buffering effect (Gisbert et al., 1999; Raji & Norouzi, 2010; Sarasquete et al., 1995). In the present study, the reduction in the volume of acidic and neutral mucins during fasting probably occurred because of similar immunological adjustments, and resuming nutrition likely stopped the inflammatory processes.

Feed intake reconfigured the intestinal performance of *P. mesopotamicus* after fasting for several days. Refeeding provided nutritional adjustments and improved intestinal absorption, thereby facilitating the consequent recovery of animal productivity. According to Py et al. (2022), fasting/refeeding protocols differ in species, duration, and intensity, which can lead to divergent results regarding compensatory growth. Furthermore, this practice involves metabolic, digestive, oxidative, and immune changes that appear to generate poorly understood physiological consequences in organisms.

## 5 CONCLUSION

Feed deprivation caused severe changes in the biometric and histomorphological parameters of pacu (*P. mesopotamicus*), leading to the observed intestinal adaptations. However, the effects of fasting were attenuated and even improved with subsequent refeeding. These results indicate that *P. mesopotamicus* is suitable for production based on fasting/refeeding management for as long as 30 days of fasting. Furthermore, this strategy is practical and sustainable for aquaculture management of *P. mesopotamicus*.

## AUTHOR CONTRIBUTIONS

**Karine Nathiele Nogueira Farias:** Conceptualization; methodology; investigation; writing – original draft. **André Luiz do Nascimento Silva:** Methodology; investigation. **Marco Shizuo Owatari:** Writing – review and editing. **Sabrina Fuzer Gonçalves:** Methodology; investigation. **Robson Andrade Rodrigues:** Conceptualization; methodology; investigation. **Cristiane Meldau de Campos:** Writing – review and editing; methodology; validation. **Lilian Franco‐Belussi:** Conceptualization; methodology; data curation; formal analysis; writing – review and editing; writing – original draft. **Carlos Eurico Fernandes:** Conceptualization; methodology; funding acquisition; investigation; writing – review and editing; formal analysis; supervision; data curation.

## FUNDING INFORMATION

This study was funded in part by the Coordenação de Aperfeiçoamento de Pessoal de Nível Superior, Brasil (CAPES; Finance Code 001). Carlos Eurico Fernandes was supported by the Conselho Nacional de Desenvolvimento Científico e Tecnológico (grant 309358/2023‐0). Karine Nathiele Nogueira Farias received a doctoral fellowship from CAPES. This study was also supported by Fundação de Apoio ao Desenvolvimento do Ensino, Ciência e Tecnologia do Estado de Mato Grosso do Sul (FUNDECT 174/2023).

## CONFLICT OF INTEREST STATEMENT

The authors declare that they have no known competing financial interests or personal relationships that may have influenced the work reported in this study.

## Data Availability

The data that support the findings of this study are available from the corresponding author, Carlos Eurico Fernandes, upon reasonable request.
